# An externally validated prognostic model for critically ill patients with traumatic brain injury

**DOI:** 10.1002/acn3.52148

**Published:** 2024-07-07

**Authors:** Yan Lu, Qiaohong Zhang, Jinwen Jiang, Yongjun Zhang

**Affiliations:** ^1^ Clinical Laboratory Affiliated Dongyang Hospital of Wenzhou Medical University 60 West Wuning Road Dongyang Zhejiang 322100 China

## Abstract

**Objective:**

Patients with traumatic brain injury (TBI) who are admitted to the intensive care unit often exhibit critical conditions; thus, early prediction of in‐hospital mortality is crucial. In this study, we aimed to develop a reliable and easily promotable model for predicting the in‐hospital mortality of critically ill patients with TBI using easily accessible indicators and validate the model using external data.

**Methods:**

Patient data from the Medical Information Mart for Intensive Care‐IV 2.2 database were used as training and internal validation sets to establish and internally validate the prognostic model. Data from the Affiliated Dongyang Hospital of Wenzhou Medical University were used for external validation. The Boruta algorithm was used for the initial feature selection, followed by univariate and multivariate logistic regression analyses to identify the final independent predictors. The predictive performance was evaluated using a receiver operating characteristic curve, calibration curve, clinical practicality decision curve analysis, and clinical impact curve.

**Results:**

This study included 3225 patients (training set: 2042; internal validation set: 874; and external validation set: 309). Ten variables were selected for inclusion in the nomogram model: age, mechanical ventilation usage, vasoactive agent usage, intracerebral hemorrhage, temperature, respiration rate, white blood cell count, platelet count, red blood cell distribution width, and glucose. The nomogram demonstrated good predictive performance in both the internal and external validation sets.

**Interpretation:**

We developed an externally validated nomogram that exhibited good discrimination, calibration, and clinical utility for predicting in‐hospital mortality in critically ill patients with TBI.

## Introduction

Traumatic brain injury (TBI) is a common cause of permanent disability and patient mortality.^1^, ^2^ Owing to falls, traffic accidents, and other causes, an estimated 70 million people worldwide experience TBI every year, which is a major public health problem.^3^ Owing to the limited resources available in the intensive care unit (ICU), patients with TBI who enter the ICU are often in a critical condition. Therefore, the early prediction of in‐hospital mortality is crucial, as it helps clinicians allocate medical resources reasonably and provide appropriate psychological expectations for patients' families.

In previous studies, researchers constructed various prognostic prediction models for TBI. For example, Matsuo et al.^4^ used machine learning technology to build a prognostic model for TBI, which showed good predictive performance; however, the sample size was small and lacked external validation. Yang et al.^5^ proposed a prognostic model to predict early mortality (within 14 days) in patients with TBI. Age, Glasgow Coma Scale (GCS) score, damage area, apolipoprotein E genotype, serum C‐reactive protein, interleukin 8 (IL‐8) levels, and Marshall computed tomography score were used as predictors. However, some of these indicators, such as the apolipoprotein E genotype, have not been widely used clinically and are not conducive to the promotion of the model on a large scale. In addition, corresponding prognostic models for traumatic subdural hemorrhage^6^ and skull fracture^7^ in patients with TBI exist. However, the disease progression of patients with TBI is highly heterogeneous, and building a prognostic model that is easy for medical workers to use and has good performance is still very challenging.

As most critically ill patients with TBI admitted to the ICU are delirious and unable to communicate and provide feedback, clinicians require a reliable and objective predictive tool to assess patients' risks and formulate treatment plans and resource allocations in the absence of direct patient feedback. In this study, we aimed to develop a reliable and easily generalizable model that can be validated with external data for predicting in‐hospital mortality in critically ill patients with TBI using easily accessible predictors. These factors include demographic parameters, GCS scores, vital signs, routine hematological test results, and clinical interventions.

## Methods

The design and reporting of this study were guided by Transparent Reporting of multivariable prediction models for Individual Prognosis or Diagnosis (TRIPOD) ‐art intelligence (TRIPOD‐AI).^8^ The TRIPOD‐AI checklist can be found in Table S1.

### Training and internal validation sets

Data were obtained from the Medical Information Mart for Intensive Care (MIMIC)‐IV 2.2 database.^9^ This database contains information on patient visits to the Beth Israel Deaconess Medical Center from 2008 to 2019, including vital signs, laboratory tests, treatment courses, diagnostic results, and other data. One author (Lu) passed a training course and received permission to extract data for research purposes (license number: 35953547). The collection of patient information from this database was reviewed by the Institutional Review Board of the Beth Israel Deaconess Medical Center, which waived the need for informed consent of the patients because of the anonymous and retrospective nature of the data.

Patients with TBI in the database were identified using both the International Classification of Diseases, Ninth Edition (ICD‐9) and the International Classification of Diseases, Tenth Edition (ICD‐10) (ICD‐9: 85,800,801,803,804; ICD‐10: S06). The exclusion criteria were (1) patients with repeat admissions for TBI besides the first admission, (2) patients with missing ICU admission information, (3) patients <18 years old, and (4) pregnant women. Patients ultimately included in the study were randomly grouped into training and internal validation sets in a 7:3 ratio.

### External validation set

Patients were recruited from the Affiliated Dongyang Hospital of Wenzhou Medical University between 2015 and 2023. The inclusion and exclusion criteria were the same as those used for the training and internal validation sets. This study was reviewed and approved by the Ethics Committee of the Affiliated Dongyang Hospital of Wenzhou Medical University (2024‐YX‐102). The committee waived the need for written informed consent because the data were anonymized and collected retrospectively.

### Data extraction

The following demographic data were obtained: age at admission, sex, duration of ICU stay, and in‐hospital survival. Body mass index, vital signs, and laboratory parameters, including temperature; heart rate; respiratory rate; white blood cell (WBC) count; red blood cell (RBC) count; platelet count; hemoglobin level; red blood cell distribution width (RDW); potassium, sodium, chloride, calcium, creatinine, albumin, and glucose levels; prothrombin time (PT); partial thromboplastin time (PTT); and international normalized ratio (INR), were collected from the patients' first recordings within the first 24 h of ICU admission. Moreover, the lowest GCS scores and intracranial injury types during the first 24 h of ICU admission were extracted. In addition, necessary life support records such as mechanical ventilation and vasoactive agent treatment were recorded.

The primary outcome of this study was the in‐hospital mortality rate of patients after ICU admission. Patients were considered survivors if no deaths were recorded in the hospital after ICU admission.

### Data preprocessing

For variables featuring missing values <30%, multiple imputation was performed on the training, internal validation, and external validation sets using the “mice” package of the R software version 4.2.0 (http://www.r‐project.org/).^10^ Body mass index and albumin were excluded due to missing values >30%. For continuous variables, <1% or more than 99% were considered outliers and winsorized using the winsor2 command in the STATA software (version 14.0; Stata Corp., College Station, TX, USA).

### Statistical analysis

Continuous variables are expressed as mean (SD) or median [IQR] using the Shapiro‐Wilk test to detect the normality of distribution. Categorical variables were expressed as numbers (%). Differences in the distribution of continuous variables between the two cohorts were compared using either a *t*‐test or a Wilcoxon test. Categorical variables were compared between the groups using the chi‐square test.

The training set was used to construct the prognostic model. First, the Boruta algorithm was used for feature selection, which is a method based on random forest to find the most important features from a given feature set and filter out features that have no significant impact.^11^ The filtered feature set was then subjected to univariate and multivariate logistic regression analyses to determine the independent predictors that could be included in constructing the predictive model. The predictive model was visualized by a nomogram model, which was constructed using the R software “rms” package.

Internal and external validation cohorts were used to evaluate the performance of prognostic models. The receiver operating characteristic (ROC) curve was plotted, and the area under the curve (AUC) was used to measure the predictive ability and discrimination of the model. The degree of calibration was evaluated using the Hosmer‐Lemeshow test (H‐L test), and the accuracy of the new model was evaluated by plotting the calibration curve to visualize the presentation of the data. In addition, the decision curve (DC) and clinical impact curve (CIC) were drawn to evaluate the clinical utility of the prognostic model.

## Results

**Figure 1:**
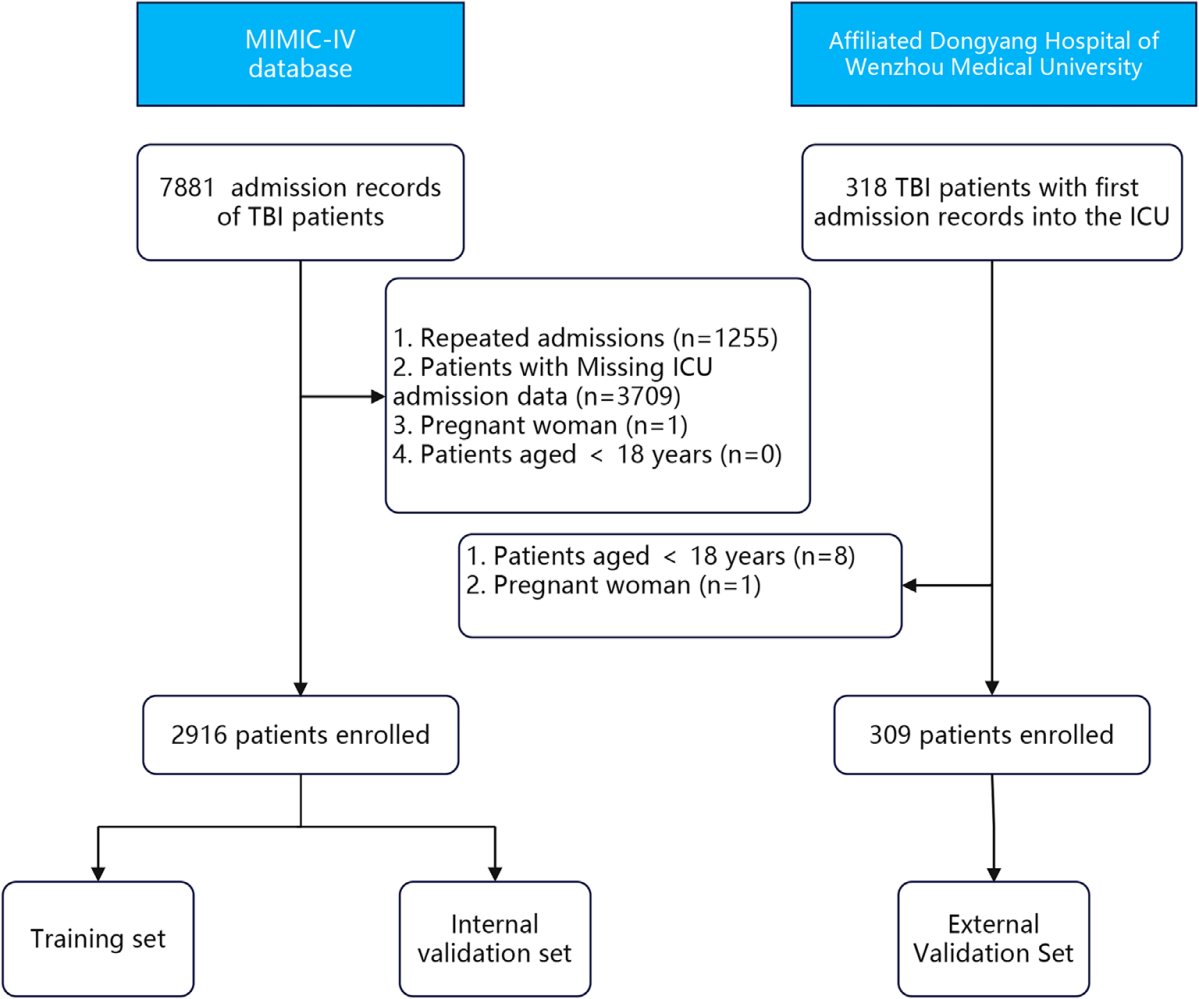
Screening process for the study population.

A total of 2916 patients from the MIMIC‐IV database and 309 patients from Dongyang Hospital of Wenzhou Medical University who were diagnosed with TBI met our selection criteria (Fig. 1). In this study, 2916 patients with TBI from the MIMIC‐IV database were randomly divided into training (*n* = 2042) and internal validation (*n* = 874) sets. Table S2 shows the distribution of all variables in the training and internal validation sets.

### Feature selection and model building

**Figure 2:**
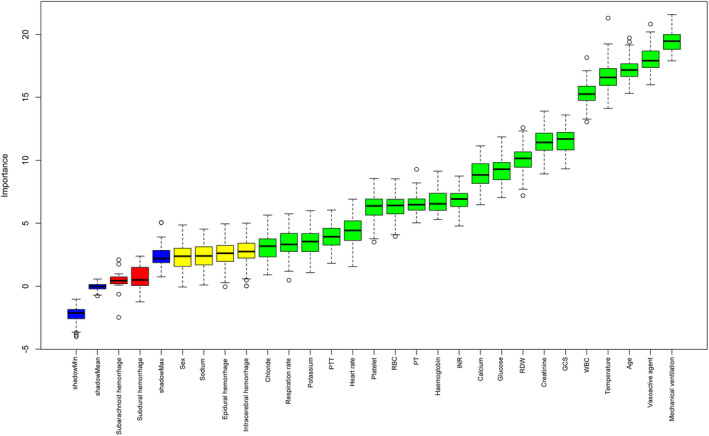
Feature selection based on the Boruta algorithm is shown. The horizontal axis represents the name of each variable, and the vertical axis represents its *Z*‐score. The box plot shows the *Z*‐score for each variable during the model calculation process. Green boxes represent the top 20 important variables, yellow boxes represent tentative attributes, and red boxes represent unimportant variables. PTT, partial thromboplastin time; RBC, red blood cell; RDW, red blood cell distribution width; INR, international normalized ratio; PT, prothrombin time; GCS, Glasgow coma scale; WBC, white blood cell.

The results of the feature selection based on the Boruta algorithm for the training set are shown (Fig. 2). Sorted by the *Z*‐value, subdural and subarachnoid hemorrhages were considered insignificant variables and were not included in the logistic regression analysis. Univariate and multivariate logistic regression analyses identified age, mechanical ventilation use, vasoactive agent use, intracerebral hemorrhage, temperature, respiration rate, WBC count, platelet count, RDW, and glucose as independent prognostic factors for in‐hospital mortality in critically ill patients with TBI (Table 1).

**Table 1:** Univariate and multivariate analyses of in‐hospital mortality of critically ill patients with traumatic brain injury in the training set.

Characteristic	Univariate			Multivariate		
	OR	95% CI	*P*‐value	OR	95% CI	*P*‐value
Age	1.02	1.01–1.03	<0.001	1.04	1.03–1.05	<0.001
Sex	0.84	0.64–1.09	0.180			
Glasgow Coma Scale score	0.92	0.88–0.96	<0.001	0.97	0.92–1.02	0.243
Mechanical ventilation	3.38	2.59–4.41	<0.001	3.31	2.36–4.65	<0.001
Vasoactive agent	6.68	4.97–8.97	<0.001	3.41	2.31–5.04	<0.001
Intracerebral hemorrhage	1.87	1.28–2.75	0.001	1.94	1.23–3.04	0.004
Epidural hemorrhage	1.12	0.81–1.55	0.495			
Temperature	0.57	0.48–0.68	<0.001	0.81	0.67–0.99	0.038
Respiration rate	1.08	1.05–1.11	<0.001	1.04	1.01–1.07	0.023
Heart rate	1.01	1.00–1.02	0.002	1.00	1.00–1.01	0.385
White blood cell count	1.10	1.07–1.12	<0.001	1.09	1.05–1.12	<0.001
Red blood cell count	0.61	0.51–0.74	<0.001	1.07	0.72–1.58	0.754
Platelet count	1.00	0.99–1.00	<0.001	1.00	0.99–1.00	0.002
Hemoglobin	0.83	0.78–0.89	<0.001	1.01	0.88–1.16	0.906
Red blood cell distribution width	1.29	1.22–1.37	<0.001	1.21	1.11–1.31	<0.001
Potassium	1.21	0.95–1.54	0.121			
Sodium	1.02	0.99–1.06	0.153			
Chloride	1.03	1.00–1.06	0.023	1.01	0.98–1.05	0.351
Calcium	0.73	0.62–0.85	<0.001	1.03	0.85–1.26	0.749
Creatinine	1.53	1.34–1.75	<0.001	1.21	1.00–1.47	0.051
Glucose	1.01	1.01–1.01	<0.001	1.00	1.00–1.01	0.001
Prothrombin time	1.18	1.13–1.22	<0.001	1.08	0.88–1.32	0.484
Partial thromboplastin time	1.06	1.04–1.07	<0.001	1.02	0.99–1.04	0.154
International normalized ratio	4.45	3.11–6.37	<0.001	0.82	0.11–6.33	0.852

CI, confidence interval; OR, odds ratio.

**Figure 3:**
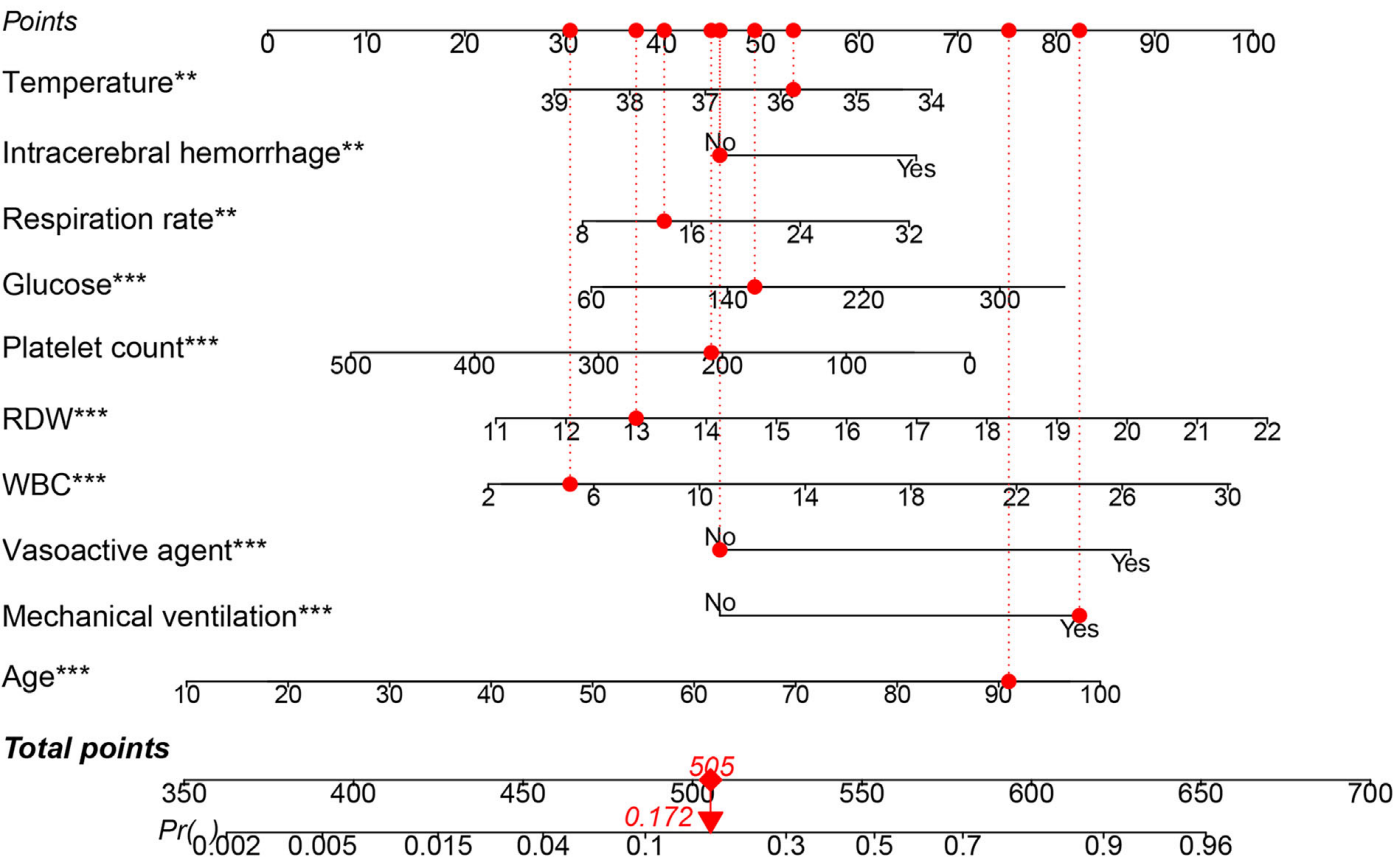
Nomogram model for predicting in‐hospital mortality in critically ill patients with traumatic brain injury (TBI). When using the nomogram, a vertical line upwards is drawn for each variable to obtain the corresponding points. The total is obtained by summing up the points of each variable. The predicted probability corresponding to the total points on the bottom scale represents the in‐hospital mortality rate of critically ill patients with TBI. RDW, red blood cell distribution width; WBC, white blood cell.

Based on the results of the feature screening, we constructed a nomogram to predict in‐hospital mortality in critically ill patients with TBI (Fig. 3).

### Internal validation

**Figure 4:**
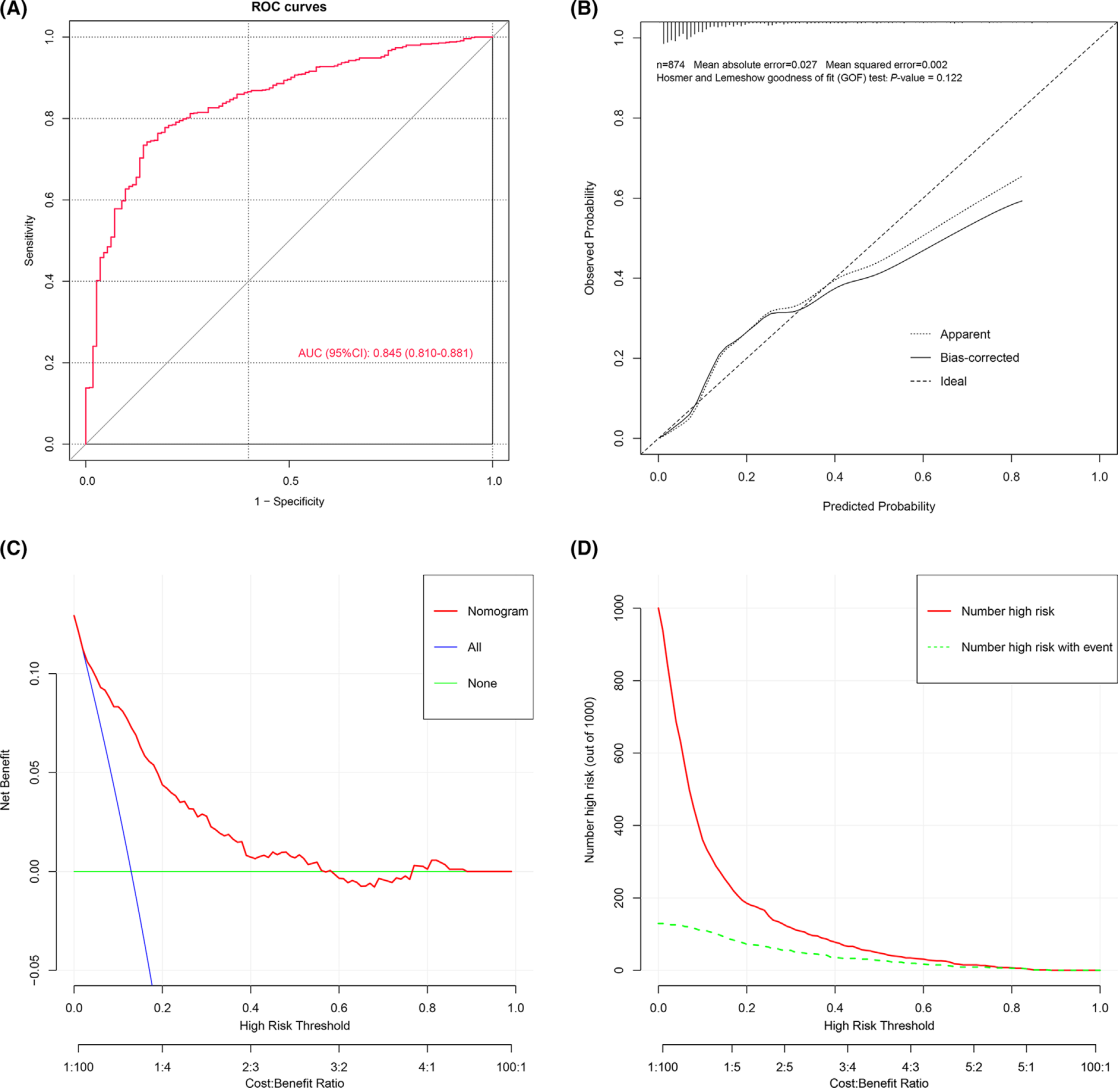
Evaluating the performance of the prognostic model in the internal validation cohort. (A) Receiver operating characteristic curve analysis of the nomogram. (B) Calibration curve analysis of the nomogram. (C) Decision curve analysis of the nomogram. (D) Clinical impact curve analysis of the nomogram.

In the internal validation set, the AUC of the nomogram model was 0.845 (95% CI: 0.810–0.881) (Fig. 4A), indicating a good predictive ability. The calibration plot of the nomogram model is shown in Figure 4B. The P value of the H‐L test was >0.05, indicating that the model had good fitting ability. DC analysis results showed that in the range of the threshold probability of 0.04–0.50, the clinical net benefit of intervention according to the predicted probability of the model was higher than that of no intervention (none) and uniform intervention (all) (Fig. 4C). According to the CIC results, when the risk threshold was greater than 0.2, the determination of the population at high risk of in‐hospital mortality in patients with TBI by the model was highly matched to the population in which death actually occurred, and the clinical prediction efficiency was high (Fig. 4D).

### External validation

**Figure 5:**
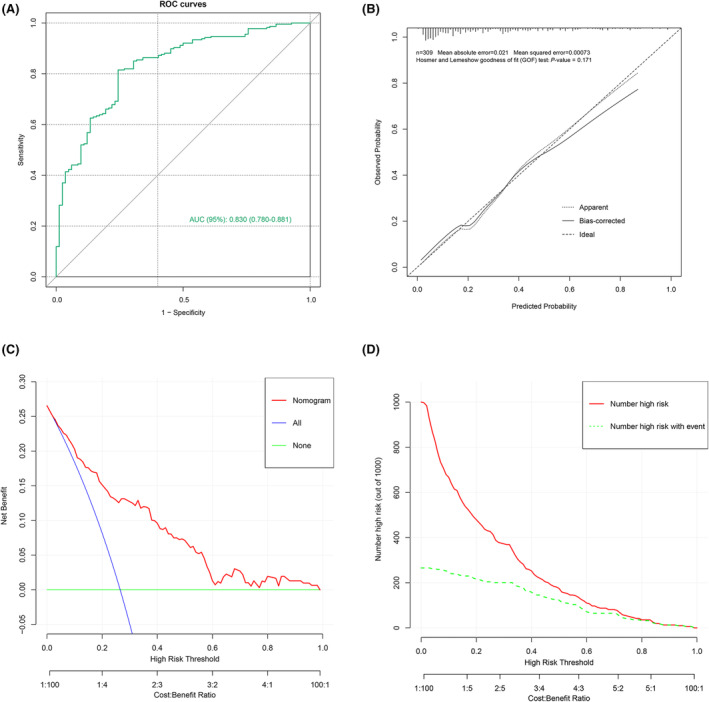
Evaluating the performance of the prognostic model in the external validation cohort. (A) Receiver operating characteristic curve analysis of the nomogram. (B) Calibration curve analysis of the nomogram. (C) Decision curve analysis of the nomogram. (D) Clinical impact curve analysis of the nomogram.

A total of 309 patients from Dongyang Hospital affiliated with Wenzhou Medical University (Zhejiang, China) from 2015 to 2023 were used as external validation sets to validate the model. Table S3 shows the comparison of variables between training and external validation sets. In this set, the discriminative performance of the nomogram model decreased slightly, with an AUC of 0.830 (95% CI: 0.780–0.881) (Fig. 5A). Both the H–L test and calibration curves showed that the predicted probabilities of the nomogram model were not significantly different from the actual probabilities, suggesting that the model was well corrected in the external validation set (Fig. 5B). The DCA and CIC curves demonstrated that the clinical interventions guided by the nomogram model were also beneficial in the external validation set (Fig. 5C, D).

## Discussion

Based on the publicly accessible MIMIC‐IV database, which is a comprehensive research database for patient medical data, we identified age, mechanical ventilation use, vasoactive agent use, intracerebral hemorrhage, temperature, respiration rate, WBC count, platelet count, RDW, and glucose as independent prognostic factors for in‐hospital mortality among critically ill patients with TBI. Using these 10 predictive variables, we constructed a nomogram model to assess the risk of in‐hospital mortality among critically ill patients with TBI. The internal validation set successfully demonstrated good discrimination, calibration, and clinical utility of the model. Furthermore, using an external validation set, we confirmed the satisfactory accuracy and stability of the nomogram model, thereby fully demonstrating its widespread applicability.

Critically ill patients with TBI admitted to the ICU differ significantly from those with normal TBI in terms of illness severity, treatment and management strategies, and prognosis.^12^, ^13^, ^14^ Prognostic predictions for critically ill patients with TBI can assist doctors in gaining a more comprehensive understanding of the patient's condition, thereby enabling the development of more individualized treatment plans. In this study, in‐hospital mortality was used as a prognostic outcome, reflecting the overall condition of patients during their hospital stay. During inpatient treatment, vital signs, changes in condition, and treatment responses can be closely monitored and evaluated. Therefore, in‐hospital mortality provides a relatively comprehensive indication of patient survival status during treatment, aiding in understanding the effectiveness of treatment measures and the likelihood of patient recovery.

Advanced age is generally considered a marker of poor prognosis in critically ill patients.^15^, ^16^, ^17^ As a persons' age increases, their physiological function and recovery capacity decline, as does the tolerance and repair capacity following brain injury. Therefore, older adult patients with TBI are usually more prone to complications and have a poor prognosis. Critically ill patients with TBI often require mechanical ventilation to support their respiratory function. Moreover, TBI patients have a higher risk of central nervous system damage, which affects the regulation of the respiratory center and leads to abnormal respiratory rate.^18^ When patients require mechanical ventilation, it usually indicates that a patient's respiratory function is severely impaired, requiring external equipment to maintain life. Mechanical ventilation may also increase the risk of infection and other complications, thereby affecting patient prognosis.^19^ In patients with TBI, the use of vasoactive agents may be due to unstable blood pressure or circulatory dysfunction, which increases the risk of cardiovascular events and death. Traumatic intracerebral hemorrhage can directly damage brain tissue, leading to neurological deficits, and has a high incidence of hemorrhagic progression, which may result in poor prognosis for TBI patients.^20^, ^21^ Patients with TBI often experience disturbances in thermoregulation. Consistent with previous findings, hypothermia was a poor prognostic factor in patients with brain injury.^7^ WBC count is an important indicator of the infection status and inflammatory response of patients. Multiple studies have shown that an elevated WBC count may indicate the exacerbation of infection or an inflammatory response in critically ill patients, which may worsen the condition of the patient and increase the risk of death.^22^, ^23^, ^24^ Platelets play crucial roles in hemostasis and coagulation. Patients with TBI often experience coagulation dysfunction, and a decrease in platelet count can lead to uncontrollable bleeding, increasing the risk of intracranial hemorrhage and other hemorrhagic complications, thereby affecting prognosis.^25^, ^26^ An abnormally elevated RDW not only indicates the presence of anemia or malnutrition in patients but also suggests that it may be a response to inflammation, increasing the risk of poor prognosis in patients with TBI.^27^, ^28^ Finally, acute hyperglycemia after TBI is a common symptom in patients with severe TBI. Clinical research consistently suggests a close relationship between acute hyperglycemia and poor prognosis.^29^, ^30^

Using extensive data from the MIMIC‐IV database, this study conducted a retrospective analysis of a large cohort to delve deeply into the various risk factors that affect the in‐hospital mortality rate of critically ill patients with TBI. Consequently, we successfully developed a nomogram model in which predictive variables could be conveniently obtained within 24 h after ICU admission, providing a timely and effective reference for clinical decision‐making. Significantly, we overcame the common issues of insufficient external validation and small sample sizes in previous studies, thereby ensuring the reliability and stability of our model through rigorous external validation and a sufficiently large sample size.

This study has some limitations. First, significant differences existed in the demographic characteristics and medical practices between patients in the training and external validation cohorts. Second, because this was a retrospective study, selection bias was inevitable. Further prospective studies are required to improve the level of evidence to support our findings. In addition, although this study included as many clinical factors and laboratory indicators as possible, some risk factors for poor prognosis may not have been determined due to the absence of or difficulty in obtaining significant variables such as albumin, body mass index, and radiological features. Finally, we acknowledge the significant heterogeneity of patients with TBI; whether subgroup analysis can improve the accuracy of patient prognosis prediction is worth further exploration in the following study. Nevertheless, our model demonstrated a satisfactory performance in predicting in‐hospital mortality in the internal and external validation sets.

Based on the MIMIC‐IV database, we developed a nomogram to predict the in‐hospital mortality rate of critically ill patients with TBI, which was validated using internal and external datasets. The nomogram exhibited good discrimination, calibration, and clinical utility in predicting the in‐hospital mortality rate in critically ill patients with TBI.

## Author Contributions

YL contributed to data collection and analysis. QZ contributed to the research concept and design. JJ contributed to the formal analysis and methodology. YZ contributed to the resources and supervision. All authors contributed to the manuscript drafting and approved the submitted version.

## Funding Information

No funding information provided.

## Conflict of Interest

The authors have no conflict of interest to declare.

## Supporting information


**Table S1.** The checklist of TRIPOD‐AI.
**Table S2.** Baseline characteristics of critically ill patients with traumatic brain injury in the training and internal validation sets.
**Table S3.** The comparison of characteristics in the training and external validation sets.

## Data Availability

The data that support the findings of this study are available from the corresponding author upon reasonable request.
